# Worldwide trend in research on *Candida albicans* and cancer correlations: a comprehensive bibliometric analysis

**DOI:** 10.3389/fmicb.2024.1398527

**Published:** 2024-05-23

**Authors:** Shiqin Tang, Yanyan Xu, Xiaojing Li

**Affiliations:** ^1^School of Clinical Medicine, The Hebei University of Engineering, Handan, China; ^2^Affiliated Hospital of Hebei University of Engineering, Handan, China

**Keywords:** *Candida albicans*, cancer, tumour, bibliometric analysis, visualization techniques

## Abstract

**Objective:**

*Candida albicans* (*C. albicans*), an opportunistic pathogen, is implicated in the carcinogenesis of various cancers, thereby significantly impacting human health. This study conducts an in-depth analysis of the prevailing research dynamics concerning the relationship between *C. albicans* and cancer over the past decade, offering a comprehensive overview of the knowledge structure and emerging focal points in this field through bibliometric scrutiny.

**Methods:**

A methodical quantitative and visual scrutiny of pertinent literature from the Web of Science Core Collection (WoSCC) spanning the previous decade was carried out employing VOS Viewer and CiteSpace software.

**Results:**

From January 1, 2014, to January 1, 2024, a comprehensive corpus of 1,259 articles was delineated. Prominent research institutions included the Egyptian Knowledge Bank, Cairo University, and King Saud University. The top three prolific countries were the United States, China, and India. Among the authors, Mohamed, Gehad G., Mahmoud, Walaa H., and Netea, Mihai G., emerged as the most prolific, with Pfaller, Ma being distinguished as the most frequently cited author. The journal Molecules published the highest number of articles, while PLoS One had the highest citation count. Nature had the highest impact factor. The research focal points in this field encompassed the interactions between *C. albicans* and cancer, the correlation with oral cancer, the underlying mechanisms of *C. albicans* carcinogenic potential, as well as antifungal and anticancer therapies.

**Conclusion:**

This investigation constitutes a pioneering bibliometric analysis elucidating the trends and advancements in research regarding the correlation between *C. albicans* and cancer. Said analyses uncover the prevailing research focal points and trends, offering insightful guidance for subsequent inquiry in this domain.

**Systematic review registration:**

https://www.webofscience.com/wos/woscc/summary/df33afba-f843-41e8-b932-cb3678eb8243-e92e7316/relevance/1

## 1 Introduction

Cancer ranks as a preeminent health challenge faced by a vast swath of the population over the course of their lives. Currently, the interplay between cancer and the human microbiota commands significant scholarly interest (Zhang et al., 2023). For an extensive duration, fungal infections, predominantly those engendered by *Candida albicans*, have presented a severe menace to human well-being, especially within immunocompromised communities. While *C. albicans* typically resides as a commensal fungus within the host, eliciting no detriment, its transformation into a pathogenic entity occurs exclusively in individuals with compromised immunity. Among cancer patients exhibiting diminished immune functionality, the probability of transition from mucosal to systemic infections attributed to *C. albicans* escalates markedly (Leerahakan et al., 2022). As a result, an expanding corpus of research is presently focused on elucidating the mechanisms by which *C. albicans* promotes carcinogenesis. Contemporary findings indicate that *C. albicans* might accelerate cancer progression through the production of carcinogenic compounds, including acetaldehyde, and the activation of pro-inflammatory pathways, thereby hastening cancer development. Accordingly, a thorough investigation into the nexus between *C. albicans* and cancer harbors significant promise in advancing our comprehension of their carcinogenic interactions and in delineating precise diagnostic criteria and therapeutic strategies.

Recent literature reviews reveal that the research trajectory pertaining to *C. albicans* and cancer is increasingly attracting scholarly attention. However, to date, there exists a notable scarcity of academic inquiries into this subject within the realm of bibliometrics. Accordingly, this manuscript elects to employ the Web of Science Core Collection (WoSCC) as its principal data source. The WoSCC, esteemed as an authoritative literature retrieval platform, enjoys widespread utilization among scholars and is deemed the quintessential database for bibliometric analysis (Wu et al., 2021). This investigation leverages the WoSCC to extract pertinent literature concerning the nexus between *C. albicans* and cancer over the preceding decade. Through the adoption of two prominent visualization instruments, CiteSpace and VOS Viewer, extensively utilized within the information science territory, this analysis undertakes quantitative and visual explorations. The objective is to discern the international research landscape and forthcoming trends within the domain of *C. albicans* and cancer, thereby fostering an in-depth understanding of the field’s evolutionary trajectory. Furthermore, the study delineates principal investigators and the contemporaneous state of research, additionally providing insights into prospective research trends and opportunities within this domain.

## 2 Research methods and data sources

### 2.1 Research methods

Bibliometrics, delineated as an auxiliary branch of informatics, encapsulates a quantitative research paradigm grounded on the scrutinization of publications, citations, and textual data. Its purpose is to decipher, characterize, and analyze the architecture, dynamics, and progressions within a given discipline or area of inquiry. It facilitates the methodical arrangement and expedited examination of an extensive array of published articles, thereby acting as a potent instrument for illuminating evolutionary patterns, publication trajectories, author citation networks, and other relevant facets vital to investigating a specific topic (Li et al., 2022; Kulwatno et al., 2023). Fundamentally, the outputs of bibliometric analyses are not solely confined to descriptive statistics but also embrace the scrutiny of keywords, texts, citations, authors, institutions, and references. This multifaceted exploration delves into the frequency, pertinence, centrality, and clustering of authorial and textual datasets (Cooper et al., 2018).

CiteSpace, a Java-powered software tool, harnesses a comprehensive database repository to facilitate co-clustering analysis across disparate modules. Utilizing similarity algorithms, it renders a graphical depiction of varying temporal dimensions (Liu et al., 2019). Moreover, it enables the exhibition of pivotal information, including frequency metrics, centrality tracings, bursts of keywords or reference citations, and the superimposition of dual journal mappings. Such visualizations augment a thorough investigative analysis probing into developmental trends and inherent transformations within the research domain (Chen, 2004, 2005; Chen et al., 2012).

VOS Viewer prioritizes the development of sophisticated visualization methodologies, enabled by the strategic employment of variables including keywords, journals, authors, countries, and institutions. An array of visualization perspectives is presented, covering Label Visualization, Density Visualization, Cluster Density Visualization, and Scatter Visualization. Within each perspective, the relevance and evolution of entities within the domain are elucidated through the observation of label sizes and circle dimensions (van Eck and Waltman, 2009).

While visualization software adeptly showcases developmental trends within a research avenue, it may exhibit constraints in grasping the intrinsic nuances of scholarly discourse. Consequently, to fully apprehend the foundational principles of a scholarly work, it is critical to amalgamate the insights derived from literature perusal with the application of visualization tools. Utilizing this integrative methodology, we are better equipped to elucidate and traverse the overarching framework, evolutionary trajectory, and avant-garde directions within the realm of scientific inquiry. By judiciously navigating a plethora of scholarly texts, we can meticulously curate the most germane studies for in-depth examination and synthesis, thus enabling a thorough dissection of burgeoning trends in *C. albicans* and cancer research.

### 2.2 Data sources

**Figure 1:**
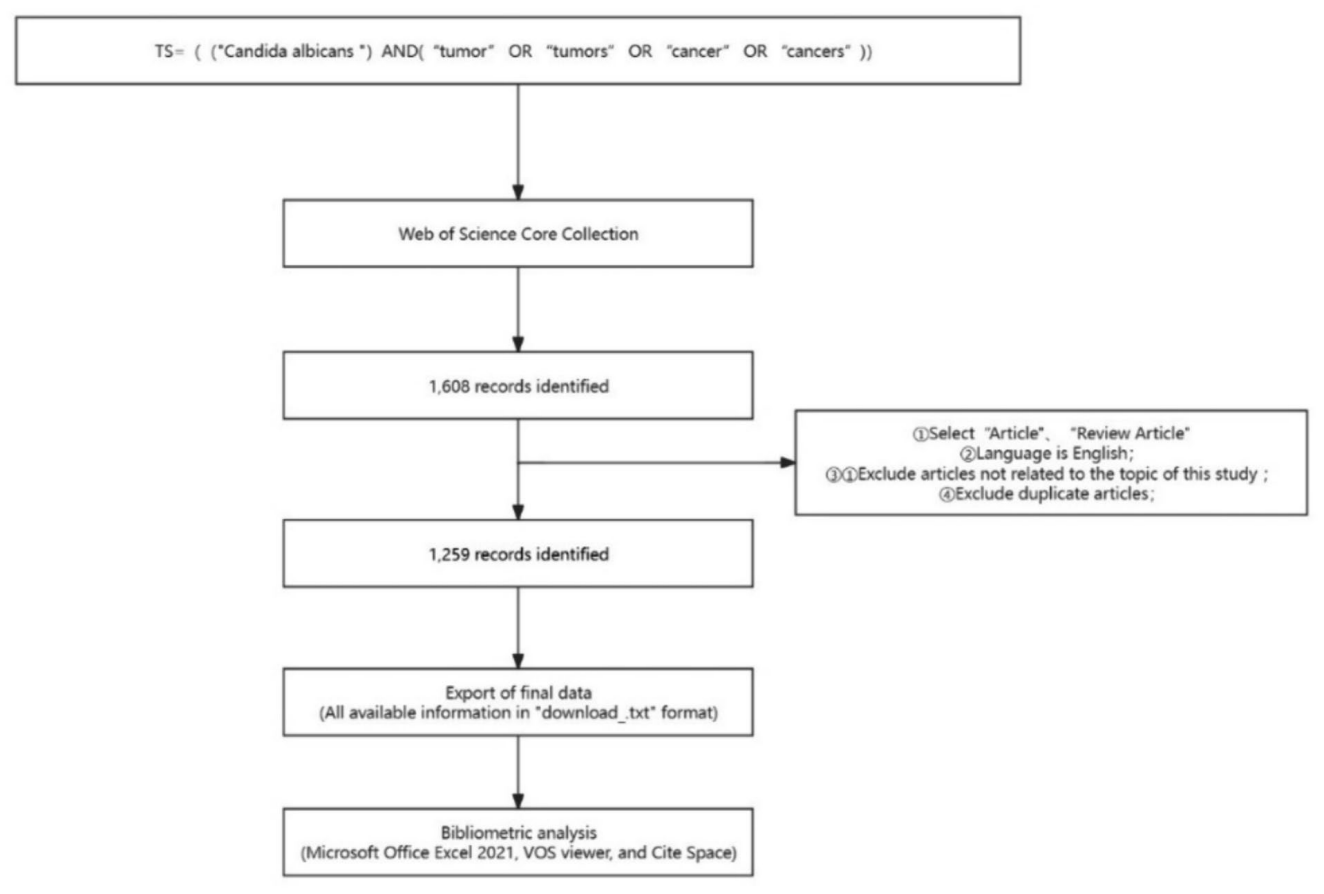
Process of publication manuscript screening.

The procurement of data from the WoSCC core database necessitated the application of rigorous research methodologies alongside a diverse array of data sources. The search query was delineated as: “TS = ((“*Candida albicans*”) AND (“tumor” OR “tumors” OR “cancer” OR “cancers”)).” To sharpen the focus of the search outcomes, solely articles and review articles penned in English were selected. A designated timeframe was determined, extending from January 1st, 2014 to January 1st, 2024. Importantly, the data for this retrieval endeavor was amassed on February 26th, 2024, a date purposively chosen to lessen any potential biases engendered by daily updates to the database. Exclusively literature directly pertinent to the thematic nucleus of this study was incorporated, whereas any superfluous publications were rigorously excluded. Following the initial retrieval phase, 1,608 articles were garnered. Nevertheless, to guarantee the precision and pertinence of the gleaned data, potential replicates and discordances relating to the central theme were rectified via a pre-processing phase, utilizing the previously delineated exclusion criteria. Following a thorough examination of each manuscript, a culled collection of 1,259 articles was adjudicated as valid. The inclusive dossiers of these manuscripts, encompassing their titles, authors, institutional affiliations (research institutions, universities, hospitals), abstracts, journals, publication dates, and references, were archived in TXT format under the compilation labeled “Full Paper Records and References.” Subsequently, the extracted literature data was assimilated into an Excel database for ensuing analysis (Figure 1).

## 3 Bibliometric analysis of the papers

### 3.1 Number of papers published

**Figure 2:**
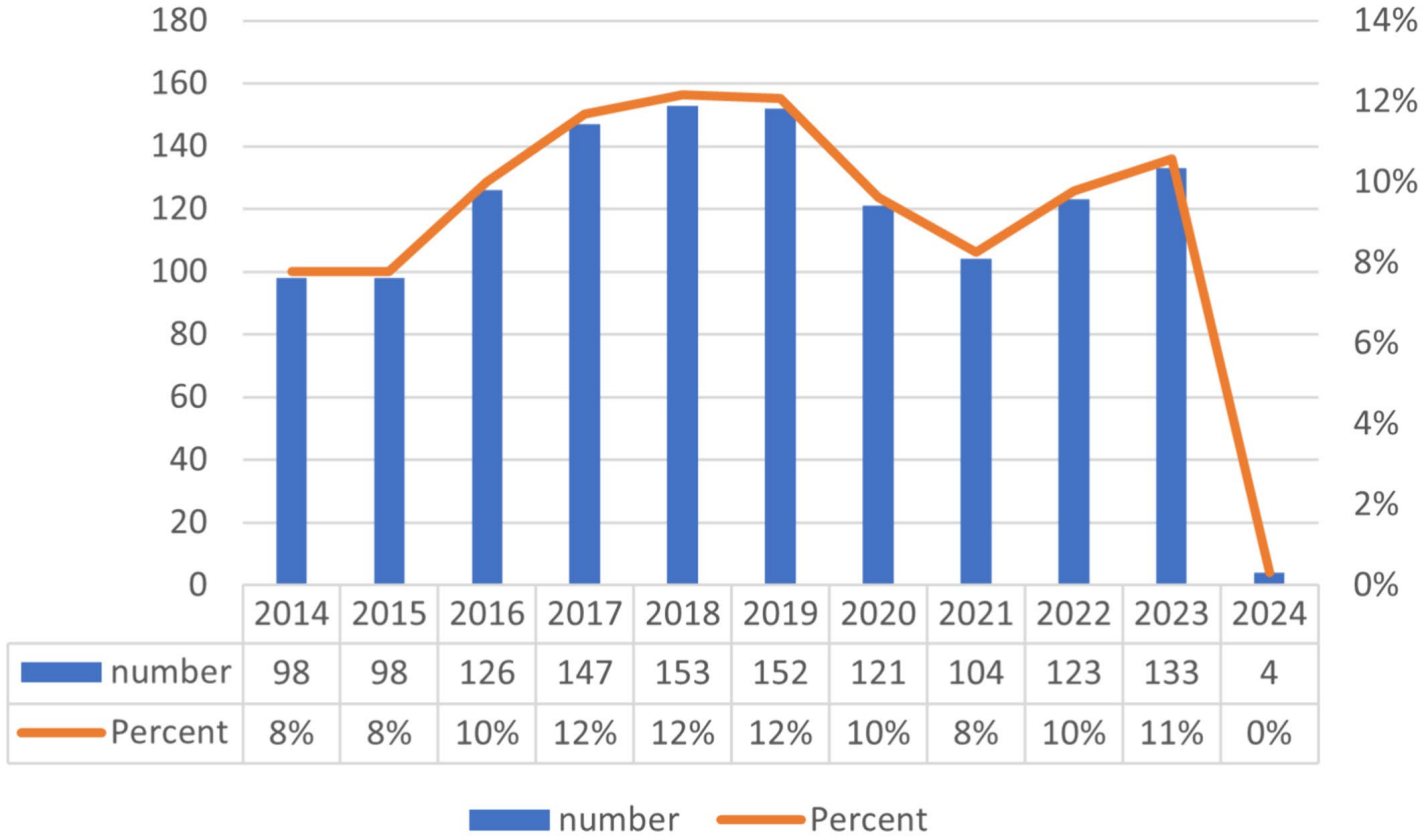
Graphically depicts the annual volume of publications over the preceding decade, elucidating the evolving dynamics of research into the interplay between *C. albicans* and cancer.

The annual fluctuations in publication volume may, to a notable degree, mirror the evolving dynamics of research themes, the intensity of academic interest, and prospective developmental trajectories. Encompassed by the ambit of this inquiry, an aggregate of 1,259 erudite articles underwent meticulous examination, emanating from 97 nations and epitomizing the collective endeavor of 7,555 authors in affiliation with 2,016 institutions. These treatises were dispersed among an eclectic array of 545 scientific periodicals, citing an extensive compendium of 62,090 citations sourced from 9,269 unique journal outlets. Figure 2 delineates the chronological dissemination of publications pertinent to *C. albicans* and cancer investigations.

Upon delving into the data, it becomes conspicuously clear that the domain under scrutiny has undergone variable publication rates throughout the previous decade. In particular, a discernible contraction in research output was noted during the years 2014 and 2015, whereas the annum 2021 registered a marginally reduced tally of publications. However, it warrants mention that the bulk of the years within the purview of this analysis have invariably sustained a publication cadence exceeding 120 scholarly articles on an annual basis. The zenith of publication activity was recorded in 2018, followed by years demonstrating a plateau in productivity trends.

### 3.2 Institutions and countries

To shed light on the nations that have significantly impacted the fields of *C. albicans* and cancer research over the preceding decade, the current investigation conducted a rigorous analysis of publication output spanning 97 countries. Table 1 illustrates the leading 20 countries and institutions, strategically distributed across continents including Asia, Europe, North America, South America, and Africa, with a marked dominance in Asia (*N* = 4) and Europe (*N* = 4) being noted. Among these nations, the United States (*N* = 233, constituting 15% of the total) and China (*N* = 212, also representing 15% of the aggregate) stand out as the principal contributing entities, closely succeeded by India (*N* = 147, accounting for 10%), Saudi Arabia (*N* = 127, contributing 9%), and Egypt (*N* = 120, representing 8%).

**Table 1:** Foremost 20 nations and institutions contributing to research in the domain of *C. albicans* and cancer.

Rank	Country	Counts	Percent	Institution	Counts	Percent
1	USA	223	15%	Egyptian Knowledge Bank(Egypt)	120	24%
2	China	212	15%	Cairo University(Egypt)	31	6%
3	India	147	10%	King Saud University(Saudi Arabia)	30	6%
4	Saudi Arabia	127	9%	Universidade Estadual Paulista(Brazil)	27	5%
5	Egypt	120	8%	Al Azhar University(Egypt)	24	5%
6	Brazil	83	6%	Council of Scientific & Industrial Research (India)	24	5%
7	Italy	53	4%	King Abdulaziz University(Saudi Arabia)	22	4%
8	Poland	53	4%	Princess Nourah bint Abdulrahman University(Saudi Arabia)	22	4%
9	Iran	51	4%	Mansoura University(Egypt)	20	4%
10	Germany	48	3%	University of Texas System(America)	19	4%
11	Japan	44	3%	Harvard University(America)	18	4%
12	Spain	41	3%	Centre National de la Recherche Scientifique (France)	18	4%
13	Turkey	40	3%	Radboud University Nijmegen(Netherlands)	18	4%
14	United Kingdom	37	3%	University System of Ohio(America)	17	3%
15	France	36	2%	Chinese Academy of Sciences(China)	17	3%
16	Netherlands	30	2%	University of California System(America)	17	3%
17	Portugal	27	2%	National Research Centre (NRC)(Egypt)	15	3%
18	Australia	24	2%	Harvard Medical School(America)	15	3%
19	Malaysia	24	2%	Jazan University(Saudi Arabia)	13	3%
20	Romania	21	1%	Islamic Azad University (Iran)	13	3%

**Figure 3:**
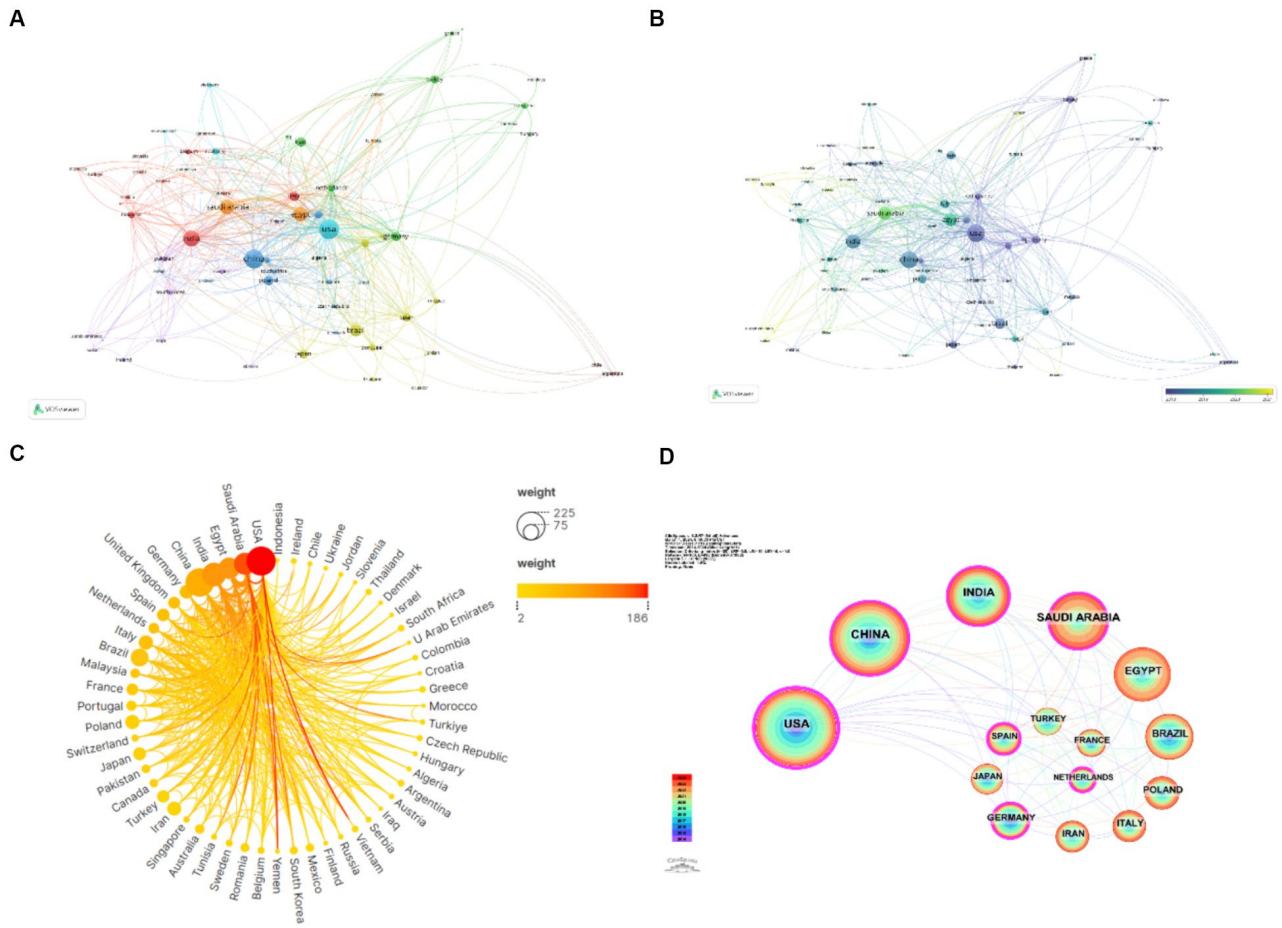
Geographic representation of research domains concerning the *Candida*-cancer nexus **(A–D)**.

Subsequent to the primary data collation, graphical representations were constructed for those countries boasting a minimum of three published articles (as depicted in Figures 3A–D). These illustrations delineate the robustness of their scholarly alliances. Enhanced line thickness between the nodes signifies an intensified rate of collaborative output among nations, whereas varied chromatic delineations denote discrete clustering configurations. The graphical data unmistakably reveals that the dispersal of authoring nations within this sector is markedly skewed, exhibiting a conspicuous top-heavy distribution. In light of the paucity of collaborative undertakings discerned among the research contingents, the encouragement of synthesis between nations and academic bodies stands out as a critical trajectory for forthcoming scholarly ventures.

Table 1 elucidates that the preeminent institutions on a global scale are as follows: the Egyptian Knowledge Bank, leading with a substantial proportion (*N* = 120, 24%), followed by Cairo University (*N* = 31, 6%), and King Saud University (*N* = 31, 6%). It is noteworthy to highlight that among the leading 20 institutions delineated, those headquartered in Egypt and the United States command the most substantial presence, each accounting for five entries on the list. This is succeeded by establishments from Saudi Arabia, which claims four spots. Followed by Saudi Arabia (*N* = 4), Brazil (*N* = 1), China (*N* = 1), India (*N* = 1), France (*N* = 1), Iran (*N* = 1), and the Netherlands (*N* = 1), underlining the diverse yet concentrated nature of excellence within this research domain.

**Figure 4:**
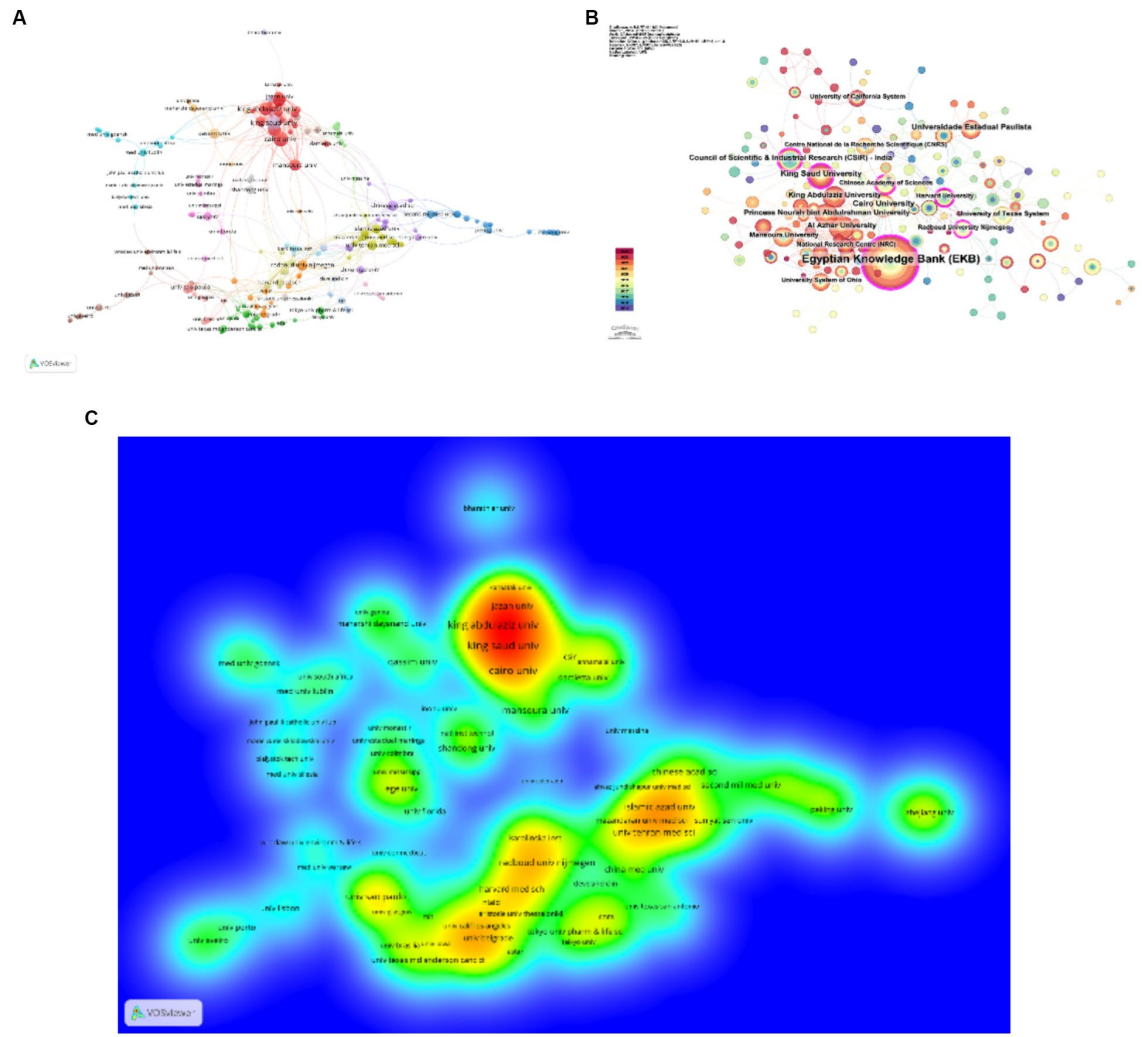
Cartographic depiction of key institutions engaged in the investigative sphere of *C. albicans* and oncology **(A–C)**.

Following this, the creation of a network visualization graph (Figures 4A–C), unveils that Cairo University is at the nexus of prolific partnerships with a cluster of esteemed institutions, including King Saud University, Al Azhar University, King Abdulaziz University, Mansoura University, Ain Shams University, and Damietta, among others. Moreover, there exists a vibrant network of collaboration among several leading global research entities such as the Agency for Science Technology and Research, Cardiff University, Georgetown University, The Johns Hopkins University School of Medicine, and The Medical University of Innsbruck, along with additional organizations. This web of interconnectedness highlights the complex and dynamic landscape of institutional cooperation spanning across continents, driving forward the boundaries of research and knowledge dissemination in the field.

### 3.3 Author analysis

**Figure 5:**
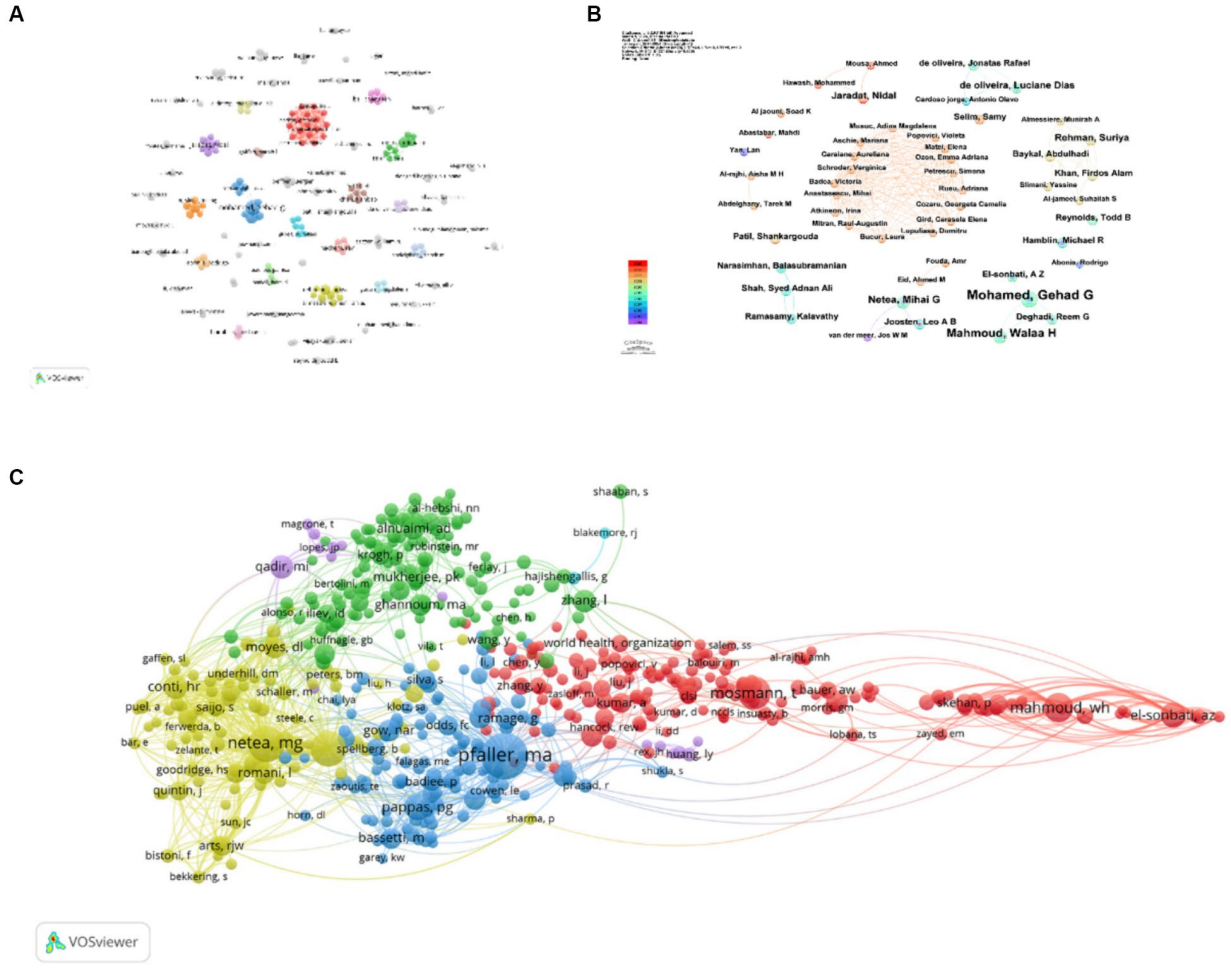
Graphical elucidation of academics engaged in the study of *C. albicans* and oncology in the previous decennium **(A,B)**. Elucidation of scholars referenced in the realm of *C. albicans* and cancer investigation within the same decennium **(C)**.

The citation frequency of a research paper is deemed a crucial indicator for evaluating its academic impact. Among prolific authors worldwide, a total of 7,555 individuals participated in studies related to *C. albicans* and cancer. Notably, the top 10 ranked authors have each published five or more articles in this field. Among this esteemed cohort, four authors garnered more than 100 citations. Pfaller, M. A. tops the list with 205 citations, followed by Netea, M. G. (121 citations), Brown, G. D. (115 citations), and Mosmann, T. (100 citations) (Table 2). Leveraging this dataset, we constructed a collaborative network diagram of authors who have published three or more papers (Figures 5A,B). Consequently, 178 authors surpassed the threshold, and we classified them into distinct clusters based on the time of their appearance, which is reflected through coloration. Each node within the graph represents an individual author, with the size of the circle denoting their publication output. The lines connecting these circles signify collaborative co-occurrences among different researchers. Notably, Mohamed, Gehad G., Mahmoud, Walaa H., and Netea, Mihai G. command the largest nodes due to their extensive contribution to relevant publications. For instance, authors such as Anastasescu, Mihai, Aschie, Mariana, Atkinson, Irina, Badea, Victoria, and Bucur, Laura exhibit close collaboration ties. Further analysis reveals that a subset of authors, consisting of 68 individuals, meets the threshold by being cited no less than 25 times. To visualize their interconnections, we produced a network graph of co-cited authors (Figure 5C). Notably, collaborations extend among different co-cited authors, including Pfaller, M. A., Ramage, G., Mosmann, T., and Badiee, P.

**Table 2:** Principal investigators and conjointly cited scholars in oncological research pertaining to *C. albicans* throughout the preceding decennium.

Rank	Authors	Document	Co-cited authors	Citations
1	Mohamed, Gehad G.	14	Pfaller, M. A.	205
2	Mahmoud, Walaa H.	10	Netea, M. G.	121
3	Netea, Mihai G.	9	Brown, G. D.	115
4	Jaradat, Nidal	7	Mosmann, T.	100
5	Rehman, Suriya	7	Mahmoud, W. H.	83
6	Hamblin, Michael R.	6	Pappas, P. G.	66
7	Joosten, Leo A. B.	6	El-Sonbati, A. Z.	60
8	Ramasamy, Kalavathy	6	Conti, H. R.	54
9	Baykal, Abdulhadi	5	Qadir, M. I.	52
10	De Oliveira, Lucianedias	5	Zhang, L.	47

### 3.4 Subject and journal analysis

Since 2016, there has been observed a steady increase in articles addressing the interplay between *Cryptococcus neoformans* and host immune responses, indicating a burgeoning interest within the scholarly community in this area of study. These articles have appeared in a spectrum of 545 journals. Table 3 exhibits the top 10 journals classified by publication count.

**Table 3:** Foremost academic periodicals by publication volume on *C. albicans* and oncological studies in the antecedent decade.

Rank	Journal	*H* index	IF(2023)	JCR	Publications	Citations	Average citation/publication
1	Molecules	149	4.6	Q2	55	885	16.1
2	Frontiers in Microbiology	135	5.2	Q2	29	549	18.9
3	Scientific Reports	213	4.6	Q2	23	480	20.9
4	International Journal of Molecular Sciences	162	6.2	Q2	19	479	25.2
5	Antimicrobial Agents and Chemotherapy	259	4.9	Q2	16	252	15.8
6	Journal of Fungi	28	4.7	Q2	16	309	19.3
7	Medical Mycology	86	2.9	Q3	16	182	11.4
8	European Journal of Medicinal Chemistry	162	6.7	Q2	15	625	41.7
9	Frontiers in Immunology	124	7.3	Q2	15	848	56.5
10	PLoS One	332	3.7	Q3	15	435	29.0

The journal Molecules emerges as the foremost in this domain, with a publication count of 55 articles (25%). These articles have accrued an impressive 885 citations, boasting an average citation rate of 16.1 citations per article, thereby securing the highest rank in this metric. Following in close succession are Frontiers in Microbiology (*N* = 29, 13%), Scientific Reports (*N* = 23, 11%), International Journal of Molecular Sciences (*N* = 19, 9%), and Antimicrobial Agents and Chemotherapy (*N* = 16, 7%). Among these, Frontiers in Immunology is distinguished by the highest impact factor, boasting an IF score of 7.3.

According to Table 4, a significant observation emerges wherein all the top 10 journals demonstrate citation counts surpassing the 600 mark. PLoS One, boasting an impressive co-citation count of 1,081, claims the highest number of citations, closely followed by Antimicrobial Agents and Chemotherapy (903 co-citations), Journal of Immunology (865 co-citations), Infect and Immunity (768 co-citations), PNAS (685 co-citations), Journal of Biological Chemistry (663 co-citations), European Journal of Medicinal Chemistry (652 co-citations), Nature (631 co-citations), Clinical Infectious Diseases (622 co-citations), and lastly Molecules (618 co-citations). Nature, notably, stands out with an astonishing impact factor of 64.8.

**Table 4:** Leading journals jointly referenced in the latest decade’s studies on the association between *C. albicans* and cancer.

Rank	Co-cited journal	*H* index	IF (2023)	JCR	Co-citations
1	PLoS One	332	3.7	Q3	1,081
2	Antimicrobial Agents and Chemotherapy	259	4.9	Q2	903
3	Journal of Immunology	372	4.4	Q2	865
4	Infect and Immunity	220	3.1	Q3	768
5	PNAS	771	11.1	Q1	685
6	Journal of Biological Chemistry	513	5.5	Q2	663
7	European Journal of Medicinal Chemistry	162	6.7	Q2	652
8	Nature	1,226	64.8	Q1	631
9	Clinical Infectious Diseases	336	11.8	Q1	622
10	Molecules	149	4.6	Q2	618

Regarding the categorization of scholarly publications, this study spans various fields, including Chemistry (21%), Pharmacology and Pharmacy (20%), Microbiology (17%), Biochemistry and Molecular Biology (15%), Immunology (8%), Infectious Diseases (7%), Biotechnology and Applied Microbiology (5%), Mycology (5%), Oncology (4%), and Research and Experimental Medicine (4%). This broad spectrum showcases the interdisciplinary nature of the research findings and highlights the wide applicability of the study’s conclusions across several domains of scientific inquiry (see Table 5).

**Table 5:** Principal research domains interconnecting *C. albicans* and malignancies over the previous ten years.

Rank	Record count	Web of science categories	% of 1,259
1	264	Chemistry	21%
2	252	Pharmacology Pharmacy	20%
3	213	Microbiology	17%
4	191	Biochemistry Molecular Biology	15%
5	95	Immunology	8%
6	87	Infectious Diseases	7%
7	57	Biotechnology Applied Microbiology	5%
8	57	Mycology	5%
9	54	Oncology	4%
10	50	Research Experimental Medicine	4%

**Figure 6:**
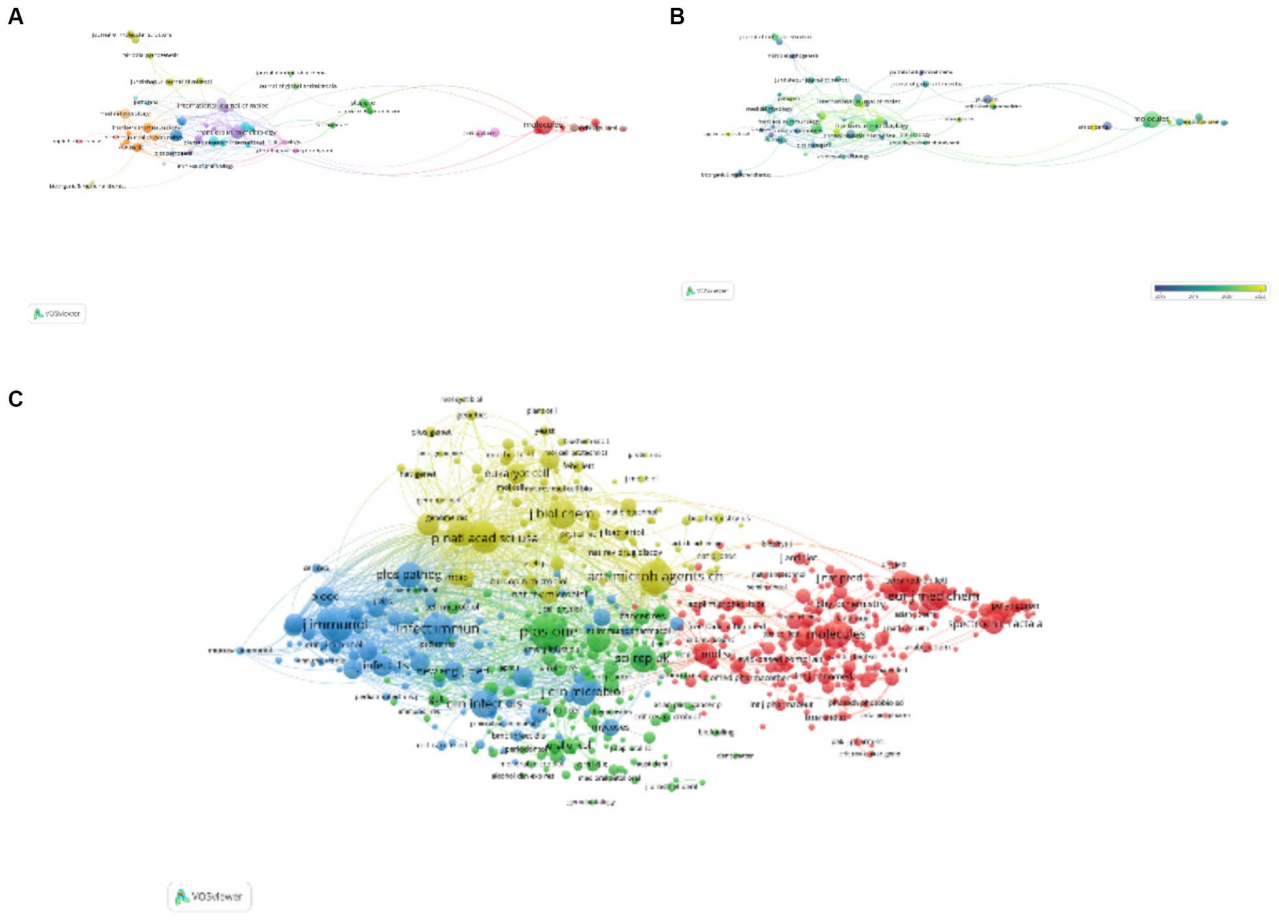
Schematic representation of collaborative frameworks within *C. albicans* and oncology research spanning the preceding decade: scholarly co-authorship matrix **(A,B)** and citation interlinkage array **(C)**.

Utilizing VOS Viewer, a curated selection of 118 journals was determined, predicated on a minimum publication threshold (*N* = 3), culminating in the conceptualization of a journal network diagram (Figures 6A,B). Of particular note, journals such as Molecules, PLoS One, and Frontiers in Microbiology were distinguished by their active citation relationships. Following this, an additional filter was applied predicated on a predefined minimum co-citation count (*N* = 20), which facilitated the selection of 677 journals for the generation of a co-citation network diagram (Figure 6C). This refined approach underscores the interconnectedness within the scholarly community, revealing the pivotal role these publications play in fostering academic discourse and development.

**Figure 7:**
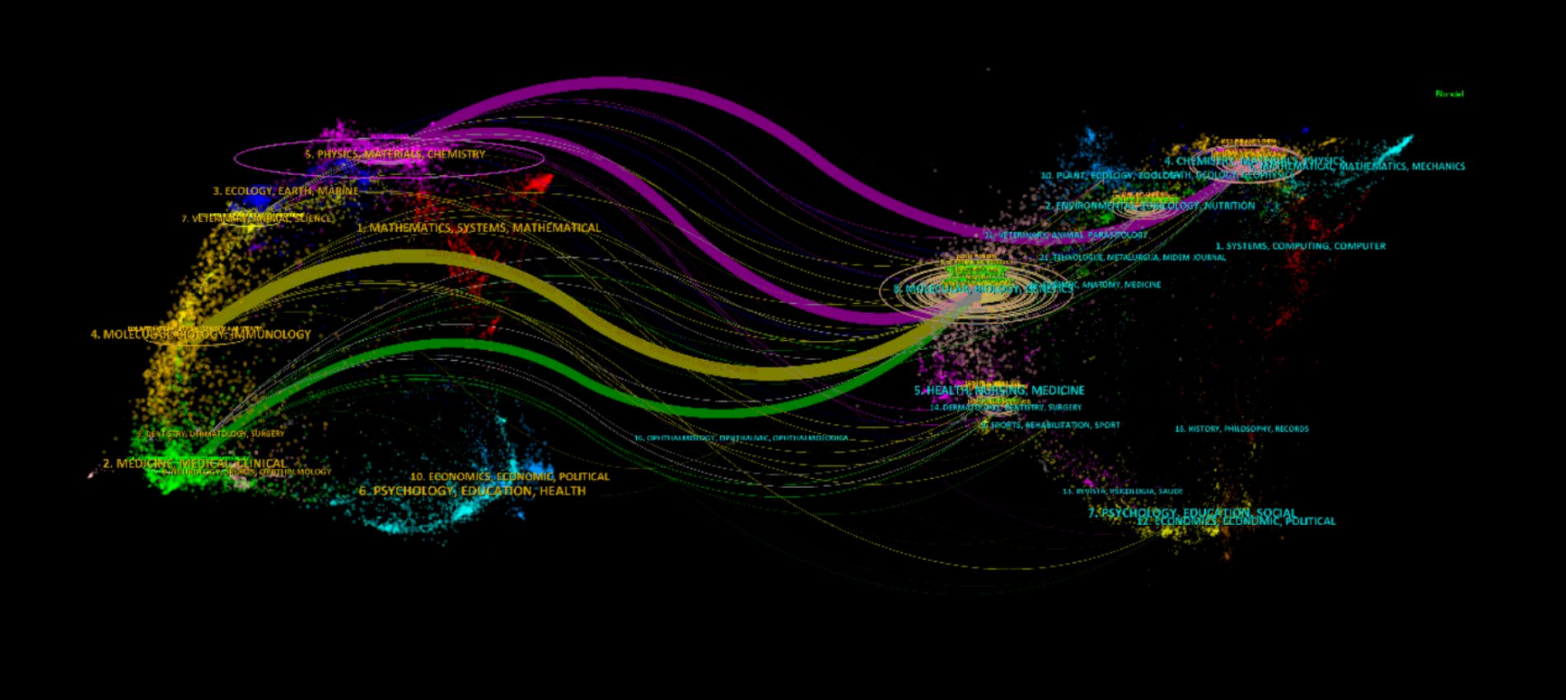
Conjoined dichotomous visualizations of periodical literature on the etiological nexus between *C. albicans* and carcinogenesis throughout the antecedent decade.

The dual mapping overlay distinctly illustrates the intrinsic relationships between the focal journal and its co-cited counterparts. On the left, citing journals are discerned, while on the right, cited journals become apparent. As delineated in Figure 7, the orange vectors symbolize the dominant citation trajectory, indicating that articles emanating from the Molecular Biology Genetics journal primarily garner citations from the Molecular Biology Immunology journal’s body of work. The green trajectories indicate citations reflecting studies from the Molecular Biology Genetics journal utilized by the Medicine Medical Clinical journal. The purple routes denote prevalent citations, where scholarly output from Molecular Biology Genetics, Chemistry, Materials, and Physics journals is frequently cited by corresponding Physics, Materials, and Chemistry journals. This mapping vividly encapsulates the multifaceted interconnections and scholarly discourse spanning across disciplines, highlighting the dynamic interchange of knowledge and research findings.

### 3.5 Co-cited references and references bursts

Within the timeframe extending from January 1st, 2014, to January 1st, 2024, a comprehensive tally of 62,090 citations was documented for research endeavors examining the correlation between *C. albicans* and cancer. Table 6 elucidates the top 10 most cited references within this sphere of study. Remarkably, all of these references achieved a threshold of a minimum of 20 citations each, while a singular publication eclipsed the distinguished landmark of 100 citations. This highlights the profound impact and significance of these scholarly works in advancing the understanding of the intricate association between *C. albicans* and oncological disorders, marking a pivotal turn in the ongoing discourse on this subject.

**Table 6:** Foremost referenced works on the correlation between *C. albicans* species and neoplasia.

Rank	Co-cited reference	Citations
1	Mosmann T. (1983). J. Immunol. Methods. 65. 55	100
2	Pfaller M. A. (2007). Clin. Microbiol Rev. 20. 133	62
3	Brown G. D. (2012). Sci. Transl. Med. 4. 12	48
4	Skehan P. (1990). J. Natl. Cancer Inst. 82. 1107	34
5	Ramirez-Garcia A. (2016). Crit. Rev. Microbiol. 42. 181	30
6	Alnuaimi A. D. (2015). Oral Oncol. 51. 139	27
7	Ghannoum M. A. (2010). PLoS Pathog. 6. 120	27
8	Bauer A. W. (1966). Am. J. Clin. Pathol. 45. 493	26
9	Mayer F. L. (2013). Virulence. 4. 119	25
10	Sokol H. (2017). Gut. 66. 1039	25

**Figure 8:**
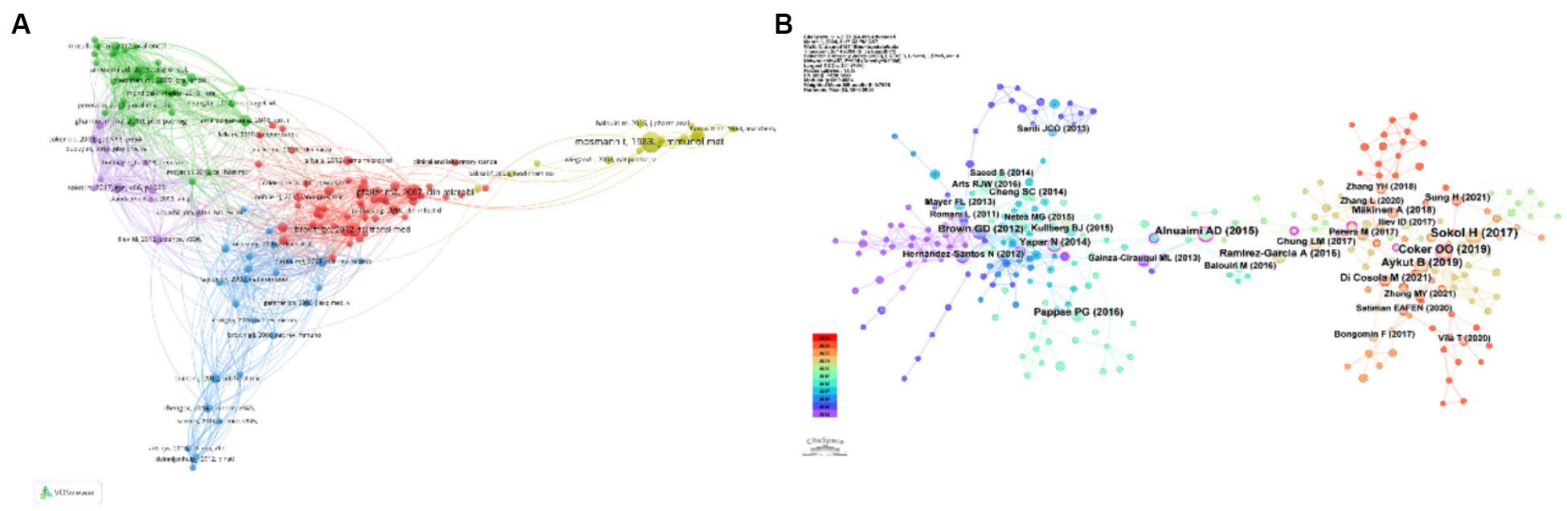
Depiction of the literature citations over the last ten years regarding the correlation between *C. albicans* and oncogenesis **(A,B)**.

Subsequently, utilizing VOS Viewer, we identified cited references that exceeded a threshold of a minimum of 10 citations to fabricate a co-citation network visualization (Figure 8A). Importantly, enlarged circles signify an elevated citation count and, consequently, an enhanced relevance of the referenced scholarly works. For illustration, distinguished references namely “pfaller ma, 2007, clin microbi,” “pappas pg., 2016, clin infect d,” and “brown gd, 2012, sci transl med” demonstrate notable co-citation associations. Employing CiteSpace, we applied a time range from 2014 to 2024, segmenting it into annual time slices, and categorized these references as the node type “cited references.” Consequently, this process resulted in the generation of a network encompassing 457 nodes (Figure 8B).

**Figure 9:**
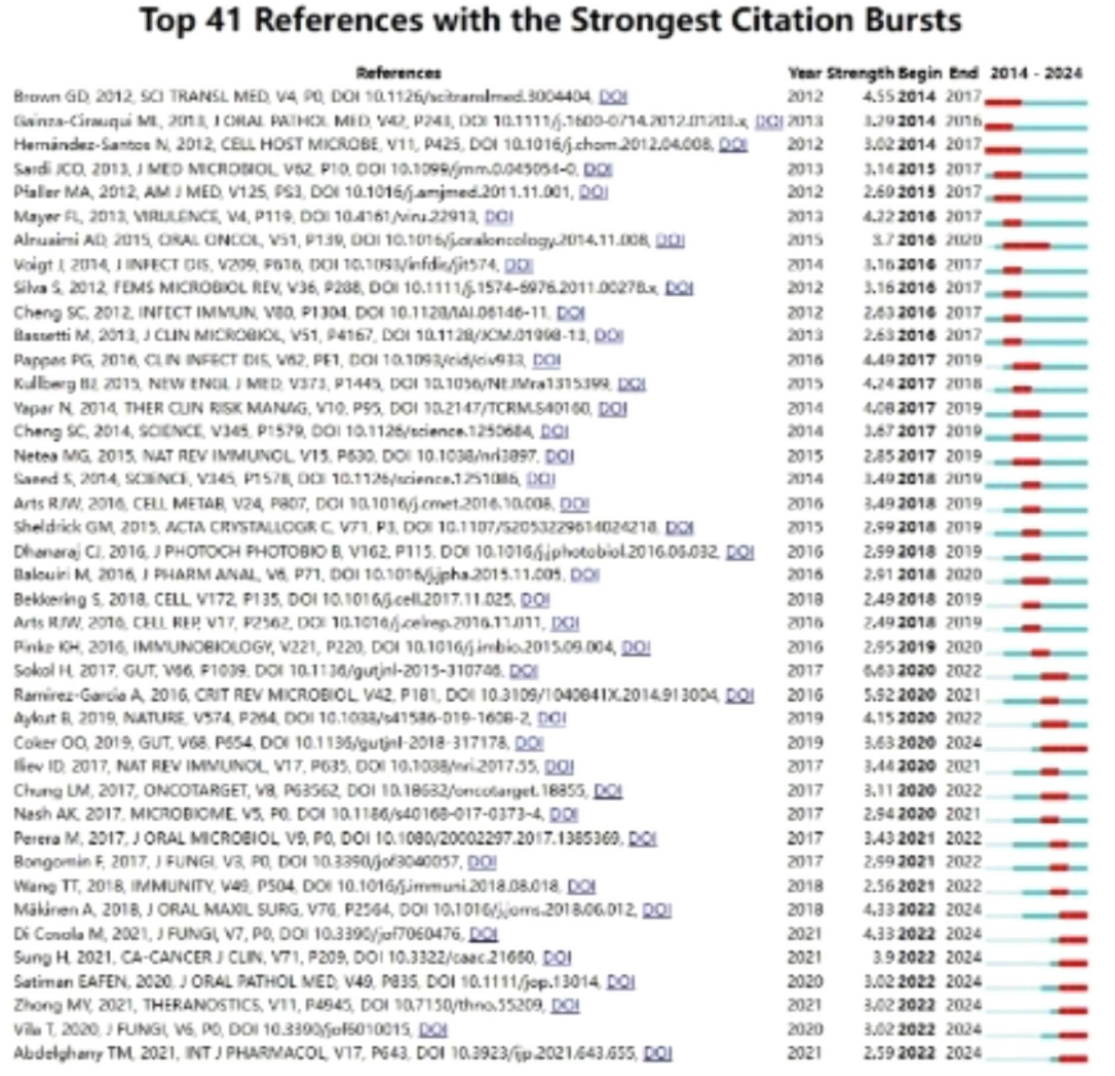
The ascendance of the principal 41 literature citations arising in the preceding decade concerning research into *C. albicans* and oncology.

Cited references within citation bursts denote reference papers frequently cited by scholars in a given field over a defined period. The repeated referencing of a collection of articles engenders a conceptual cluster (Shi et al., 2022). In the course of this investigation, employing CiteSpace, we pinpointed 41 reference papers manifesting significant citation bursts. As illustrated in Figure 9, these references are organized according to their burst initiation years. Each bar symbolizes a year, while the red lines signify the emergence of high-intensity citation bursts within a given year.

As detailed in Table 7, the reference paper exhibiting the most pronounced citation burst (intensity = 6.63) is titled “Fungal microbiota dysbiosis in IBD,” authored by Sokol et al. (2017). This citation burst endured from 2020 to 2022. Conversely, the reference paper with the second-highest citation burst intensity (5.92) bears the title “*Candida albicans* and cancer: Can this yeast induce cancer development or progression?” authored by Ramirez-Garcia et al. (2016), published in the journal “Critical Reviews in Microbiology.” The citation burst for this paper extended from 2020 to 2021.

**Table 7:** Proliferation of scholarly references for the paramount 10 articles on *C. albicans* and carcinogenesis over the recent decade.

Rank	Frequency	Centrality	Title	Journal	Author	Year
1	19	0.03	Fungal microbiota dysbiosis in IBD	*Inflammatory Bowel Disease*	Harry Sokol	2017
2	16	0.15	Enteric fungal microbiota dysbiosis and ecological alterations in colorectal cancer	*Gut Microbes*	Olabisi Oluwabukola Coker	2019
3	16	0.06	The fungal mycobiome promotes pancreatic oncogenesis via activation of MBL	*Letter*	Berk Aykut	2019
4	15	0.37	Oral *Candida* colonization in oral cancer patients and its relationship with traditional risk factors of oral cancer: a matched case-control study	*Oral Oncology*	Ali D. Alnuaimi	2015
5	12	0.02	*Candida albicans* and cancer: Can this yeast induce cancer development or progression?	*Critical Reviews in Microbiology*	Andoni Ramirez-Garcia	2016
6	12	0.14	Hidden killers: human fungal infections	*Medical Mycology*	Gordon D. Brown	2012
7	11	0.02	Clinical practice guideline for the management of candidiasis: 2016 update by the Infectious Diseases Society of America	*The Journal of Infectious Diseases*	Peter G. Pappas	2016
8	10	0.03	*Candida albicans* and oral carcinogenesis. A brief review	*Journal of Fungi*	Michele Di Cosola	2021
9	10	0.12	Epidemiology and risk factors for invasive candidiasis	*Therapeutics and Clinical Risk Management*	Nur Yapar	2014
10	10	0.06	Role of non-*albicans Candida* and *Candida Albicans* in oral squamous cell cancer patients	*Journal of Oral and Maxillofac Surgery*	Anna Makinen	2018

In Figure 9, the preeminent cited reference is delineated as “Fungal microbiota dysbiosis in IBD (Impact Factor = 24.5)” by Sokol et al. (2017). Via meticulous experimental observations, the authors identified a dominant fungal microbiota, predominantly comprised of *Candida*, within the fecal samples of individuals afflicted with Crohn’s disease. It is postulated that modifications in microbial diversity and composition could potentially play a contributory role in the genesis of IBD.

The seminal work ranked second in terms of citations, conducted by Coker et al. (2019), entitled “Enteric fungal microbiota dysbiosis and ecological alterations in colorectal cancer (IF = 24.5),” investigates the microbial dysbiosis in colorectal cancer (CRC) patients. Utilizing metagenomic sequencing and ecological analysis of fecal samples, the study uncovers that CRC is predominantly linked with fungal dysbiosis, exhibiting a more pronounced significance than bacterial-fungal co-infections.

Furthermore, in their landmark study, Aykut et al. (2019) present “The fungal mycobiome promotes pancreatic oncogenesis via activation of MBL (IF = 64.8),” arguing that fungal colonization in the gut of pancreatic ductal adenocarcinoma (PDA) patients may translocate to the pancreas. Within the host, the interaction between the mannose-binding lectin (MBL) and fungal cell wall polysaccharides is identified as the catalyst for pro-carcinogenic responses, thereby further exacerbating tumorigenesis.

In the meticulously conducted study entitled “Oral *Candida* colonization in oral cancer patients and its relationship with traditional risk factors of oral cancer: a matched case–control study (IF = 4.8),” the research team led by Alnuaimi et al. (2015) demonstrates a pronounced correlation between oral cancer and the presence of *C. albicans* colonization in the oral cavity. Moreover, they contend that genetic variations in *C. albicans* serve a contributory function in the development process of oral cancer. Moreover, within their scholarly article “*Candida albicans* and cancer: Can this yeast induce cancer development or progression?,” Ramirez-Garcia et al. (2016) explore in depth the facets of microbial infections, with a focused examination of the relationship between *C. albicans* and cancer. They offer a thorough exploration of the multifaceted evidence linking *C. albicans* with cancer promotion and metastasis. This analysis encompasses the inducement of host inflammatory responses and the genesis of secondary carcinogens. Through meticulous examination of these findings, it becomes apparent that the outbreak intensity, as reported in the 20 referenced studies, spans from 2.49 to 6.63, over durations of 1 to 4 years.

### 3.6 Hotspots and frontiers

**Figure 10:**
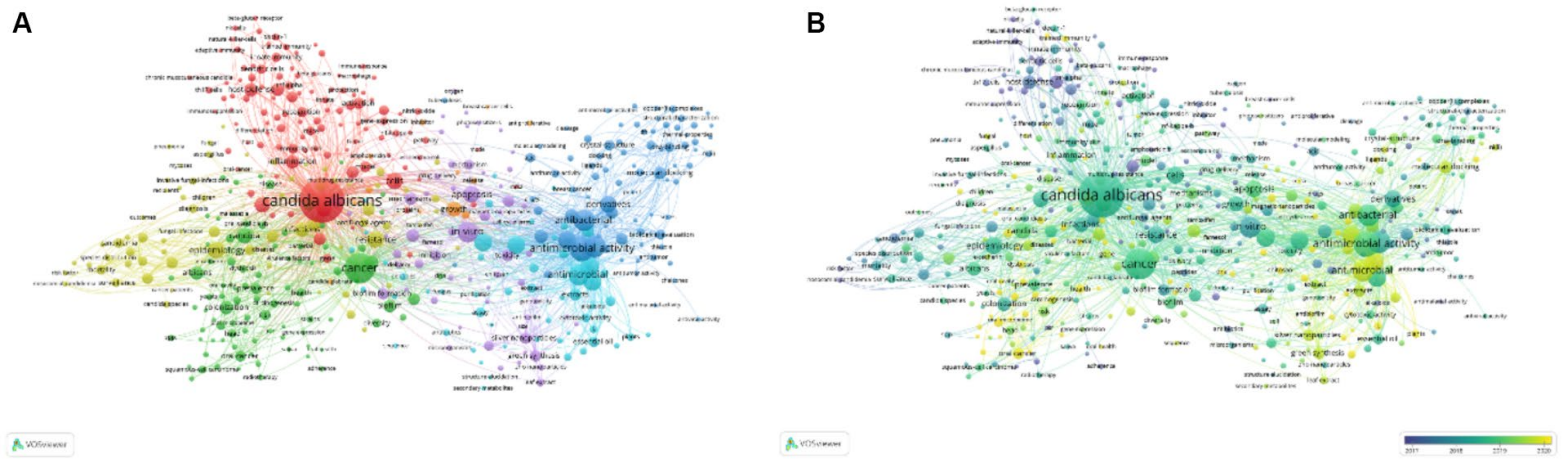
Networked depiction of salient terms pertinent to research on *C. albicans* and oncology **(A)** alongside an overlay of keyword visual representation **(B)**.

Keywords embody the essence of scholarly publications, acting as conduits through which the broader academic community may navigate the expanse of literature. The analysis of keywords in a given discipline unveils the central themes and emergent trends that seize the scholarly imagination. Within the extensive corpus of 6,782 keywords, utilizing VOS Viewer and establishing a minimum frequency threshold of five, revealed an extraordinary subset of 482 keywords that exceeded this benchmark. This select group of keywords underwent clustering analysis, with their collective connectivity strength being meticulously quantified, resulting in a visually arresting depiction of keyword clusters (Figure 10A). In the depicted schematic, the diameter of each circle is directly proportional to the keyword’s relative prominence, whereas the density of the interconnecting filaments denotes enhanced frequencies of co-occurrence among keywords. Upon the integration of a visual spectrum into the map (Figure 10B), it becomes evident that the color gradations among keyword clusters differentiate specific chronological segments. Adorned in a deep navy tone, keywords originating from the formative phases are visually set apart from their more recent counterparts, which boast a striking chartreuse hue.

**Figure 11:**
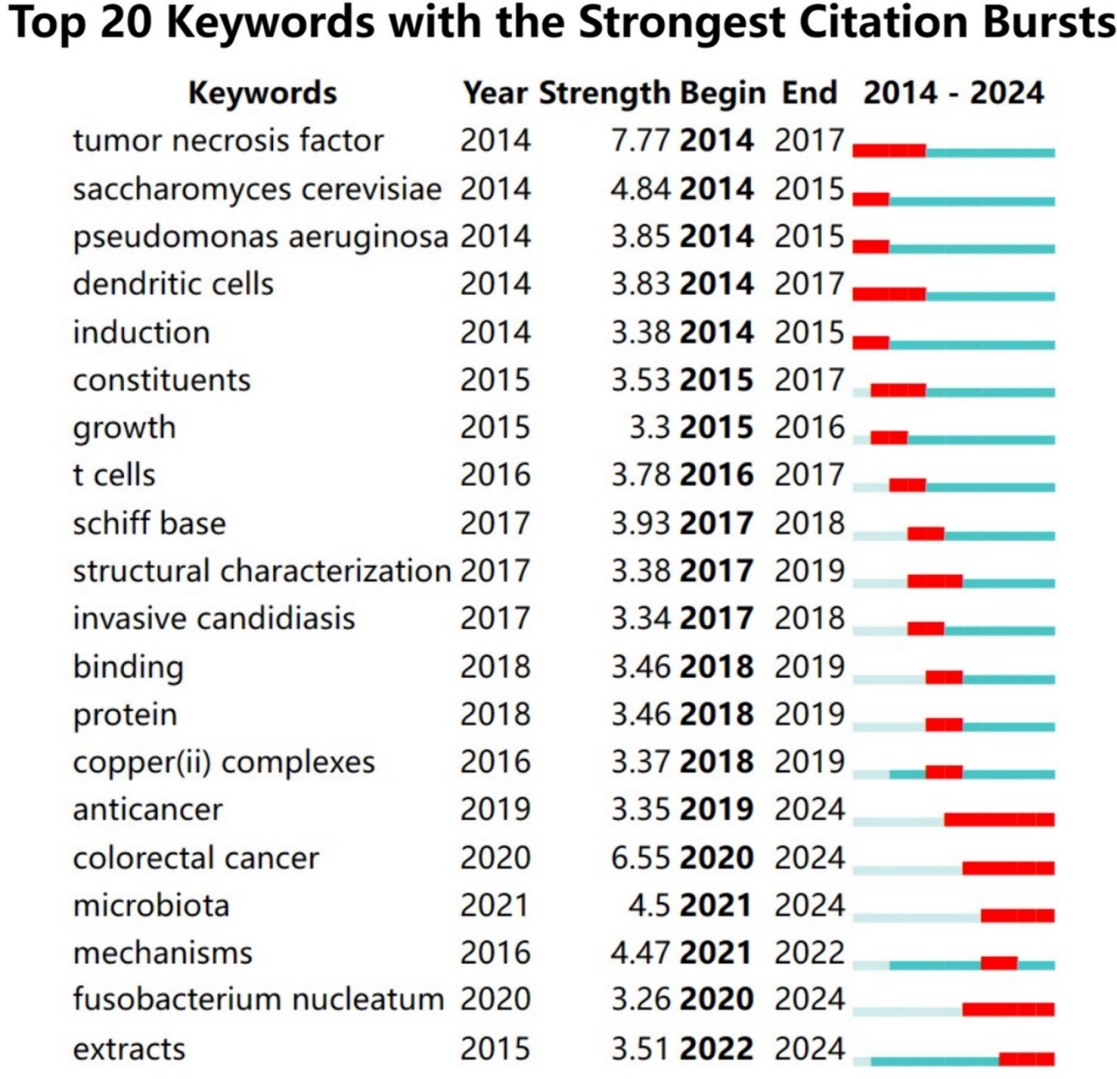
Prominence of keywords in investigative studies elucidating the association between *C. albicans* and malignancy.

Utilizing network graph visualization, an observance of key phrase eruption phenomenon was noted. Figure 11 delineates the foremost 20 key phrases extensively referenced in the domain of *C. albicans* and cancer-centric research throughout the preceding decade. Within the graph, a blue line symbolizes the chronological timeline, whereas the adjoining red fragments along this timeline indicate instances of eruption detection—marking the eruption’s inception zenith, and temporal span. Most prominently, the most pronounced key phrase eruption pertained to “tumor necrosis factor” (7.77), succeeded by “colorectal cancer” (6.55), “*Saccharomyces cerevisiae*” (4.84), “microbiota” (4.5), and “mechanisms” (4.47). Per the data, key phrases such as “tumor necrosis factor,” “*Saccharomyces cerevisiae*,” “*Pseudomonas aeruginosa*,” “dendritic cells,” “induction,” “constituents,” “growth,” “T cells,” “Schiff bases,” “structural characterization,” “invasive candidiasis,” “binding,” “protein,” and “copper (II) complexes” are categorized within the ambit of nascent inquiries. In contrast, key phrases such as “anticancer,” “colorectal cancer,” “microbiota,” “mechanisms,” “*Fusobacterium nucleatum*,” and “extracts” are in the midst of an eruption phase, hence positioning them as probable centers of contemporary scrutiny

The temporal visualization seamlessly illustrates the evolutionary trajectory of research hotspots, delineated by the selected keywords, throughout the period extending from 2014 to 2024, capturing the rise and ebb of these pivotal areas. Keywords within the same cluster are aligned on a horizontal axis, sequenced in chronological order from left to right, traversing the continuum from historical to contemporary relevance. Moreover, the proliferation of keywords within each cluster conspicuously signals the cluster’s import and its substantive contribution to the field’s progression. Utilizing CiteSpace for keyword analysis, a temporal visualization, often termed a keyword timeline, is fashioned, adeptly reflecting the zeitgeist and the fluid shifts in *C. albicans* and cancer research domains.

The appraisal of the clustering graph transpires through the assessment of Modularity *Q* and Weighted Mean Silhouette *S* metrics. The latter acts as a gauge for the cluster’s internal homogeneity. An elevated *S* value denotes heightened similarity amongst cluster modules, whereas a *Q* value exceeding 0.3 signifies a substantial partitioning framework, and an *S* value surpassing 0.5 intimates a cogent clustering methodology (Aryadoust et al., 1941). Conforming to these benchmarks, the keyword clustering analysis herein achieved a *Q* value of 0.4684, transcending the 0.3 demarcation, and an *S* value of 0.7818, eclipsing the 0.5 criterion, thus delineating a cogently justified and transparent clustering architecture.

**Figure 12:**
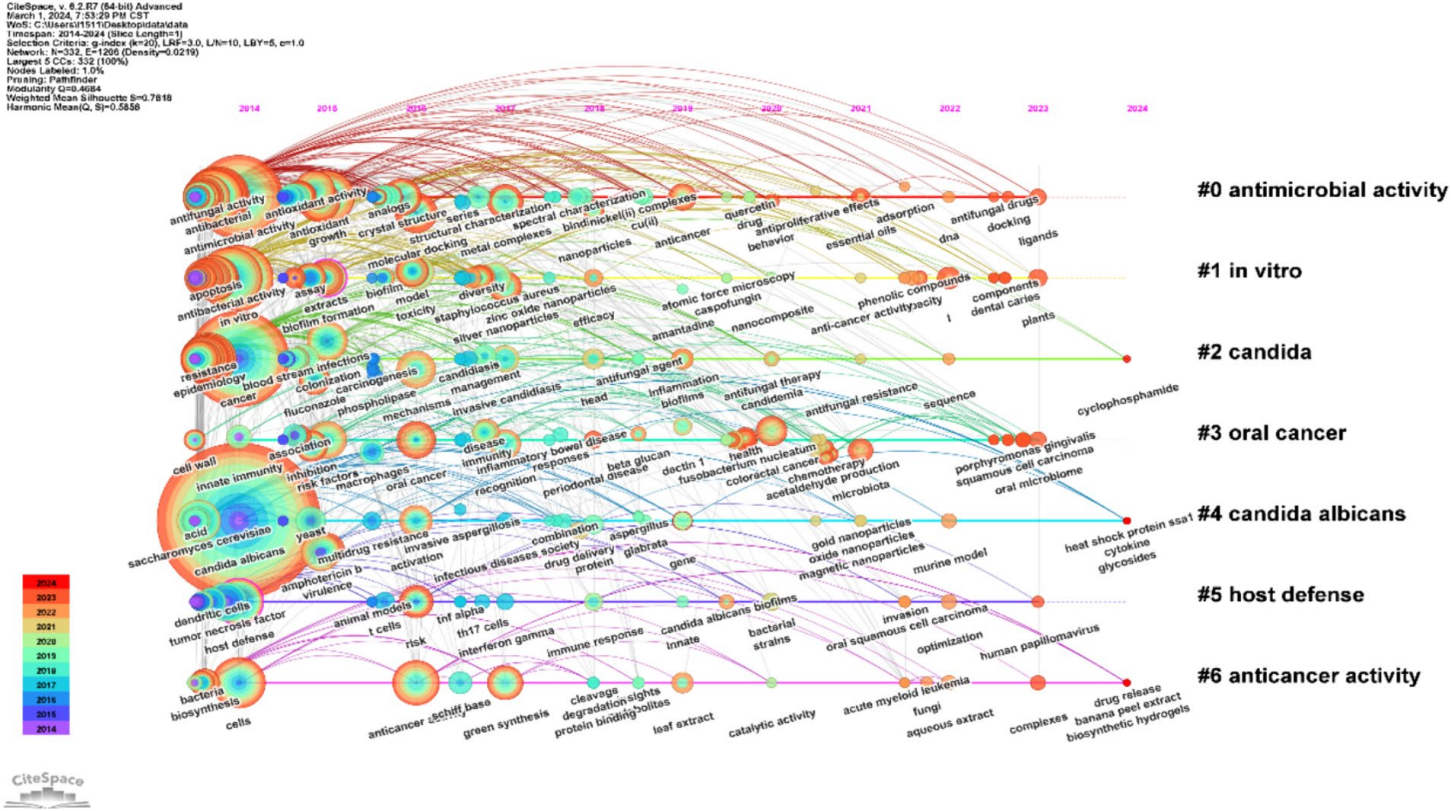
Exemplifies a chronological depiction of integral keywords related to the interrelation of *C. albicans* and neoplastic research.

Figure 12 elucidates seven unique clusters, specifically: antimicrobial activity, *in vitro*, candida, oral cancer, *C. albicans*, host defense, and anticancer activity. Each cluster embodies a singular assembly, marked by numerical identifiers like #0, #1, and #2. The broader the scope of a specific cluster, the more substantial the array of pivotal elements it encompasses (Wei et al., 2022).

## 4 Discussion

### 4.1 General information

The current inquiry necessitates the procurement of scholarly literature from the WoSCC database. Subsequent analyses deploy intricate visual methodologies via CiteSpace and VOS Viewer, thereby illuminating profound insights. This analytical purview integrates a myriad of components, including annual publication output, authorship, institutional affiliations, keywords, countries, journals, and references. Between 2014 and 2015, the research domain was discerned to be in a nascent phase. Commencing in 2016, a significant surge in publication output is observed, consistently surpassing an annual benchmark of 120 papers. The zenith of scholarly contributions materializes in 2018, indicating a sustained upward trajectory anticipated through 2024. Remarkably, in latter years, the scientific realm has exhibited an intensifying fascination with the nexus between *C. albicans* and oncology. Assessing the collaborative milieu across countries and institutions, the United States, China, and India arise as key contributors, necessitating augmented collaboration. From a holistic perspective, numerous countries demonstrate formidable collaborative endeavors, albeit some nations remain at the incipient phases of research, exhibiting restrained enthusiasm for interinstitutional collaboration. The engagement in productive partnerships proves immensely advantageous for the progression of this research field, as it facilitates the resolution of scholarly enigmas.

The Egyptian Knowledge Bank (Egypt) emerges as the foremost prolific entity in scholarly dissemination, highlighting its precocious and significant contributions to the discipline. Examination of journal hierarchies delineates Molecules (*N* = 55), Frontiers in Microbiology (*N* = 29), and Scientific Reports (*N* = 23) as possessing the paramount publication volumes within this scholarly arena. Among the referenced periodicals, PLoS One (co-citations = 1,081) stands preeminent regarding aggregate citations. Nature (IF = 64.8) secures the accolade of the most impactful periodical within this field.

This thorough exploration has revealed that within the domain of research involving Candida albicans and its associations with cancer, Gehad G. Mohamed from Egypt (*N* = 14) stands out as a distinguished contributor. Moreover, the collaboration between Mohamed and Walaa H. Mahmoud (*N* = 10) is notably profound, concentrating chiefly on deciphering the intricate chemical processes that underlie both antifungal and anticancer effects (Mahmoud et al., 2017; Mohamed et al., 2020; Khalaf et al., 2022).

In their distinguished manuscript, titled “Recurrent candidiasis and early-onset gastric cancer in a patient with a genetically defined partial MYD88 defect,” Netea, Mihai G. explores the complex interplay between *C. albicans* and gastric cancer. The study posits an increased vulnerability to recurrent *C. albicans* infections, potentially undermining the host’s immune defenses. This phenomena could conceivably be ascribed to inadequacies in IL-17 and genetic anomalies in the MYD88 gene, thus potentially causing a heightened incidence of gastric cancer (Vogelaar et al., 2016).

Furthermore, Amer et al. (2019) has penned a compendium of seven scholarly articles delving into the research of antifungal and anticancer drug efficacies. Among the top 10 cited works, a preponderance centers around fungal community, *C. albicans*, and the investigation of colorectal, oral, and pancreatic cancers. Prior research has elucidated a notably intimate correlation between *C. albicans* and oral cancer (Engku Nasrullah Satiman et al., 2020; Vadovics et al., 2021; Li et al., 2023; Wang et al., 2023), where the colonization of the oral cavity by *C. albicans* furthers oral cancer development via complex engagements with the host’s inflammatory responses. Additionally, an escalated oral presence of *C. albicans* has been implicated in the phenotypic manifestation of oral carcinogenesis. Consequently, it is evident that contemporary inquiries predominantly pivot upon deciphering the interplay between *C. albicans* and oral cancer, alongside formulating strategies to mitigate fungal advancement.

### 4.2 Hotspots and frontiers

An examination of high-frequency keywords can reveal the research landscape and evolutionary trends within a specific field of study. Leveraging this data derived from high-frequency keywords, we have examined the current research focal points. Subsequently, by exploring the temporal evolution of key terms, we have delineated the dynamics and evolution of research themes related to *Candida albicans* and cancer. The specifics are elucidated in the subsequent sections.

#### 4.2.1 Cell factors associated with *Candida albicans* and cancer

A plethora of cytokines play crucial roles in antifungal defense and also contribute indirectly to tumor promotion. Among these, the predominant CD4 T cell subset, Th17, assumes a critical role in combating *Candida albicans* infections. It is broadly recognized that Th17 cells produce IL-17, a pathway crucial for thwarting *Candida albicans* (Mengesha and Conti, 2017; Gaffen and Moutsopoulos, 2020). However, IL-17 may also inadvertently promote cancer progression indirectly by recruiting inflammatory cells such as neutrophils. Furthermore, the Th17 lineage’s production of IL-23 and IFN-γ is known to partially counteract tumorigenesis (Hao and Shan, 2006; Zamarron and Chen, 2011). Hence, during *Candida albicans* infection, alterations in host cytokines may be triggered, indirectly exacerbating the progression of cancer.

#### 4.2.2 The correlation between *Candida albicans* and cancer

*C. albicans* represents a ubiquitously distributed fungus within human hosts, constituting an integral component of the healthy human microbiota. Nevertheless, it also functions as an opportunistic pathogen, frequently engendering diseases amongst immunocompromised individuals (Ho et al., 2021). Under conditions of inflammation, *C. albicans* frequently transitions between its spore and hyphal forms. Increasingly, contemporary research posits that the dichotomous nature of this fungus may play a contributory role in carcinogenesis (Ramirez-Garcia et al., 2016). Amongst the fungi implicated in cancer, *C. albicans* stands as the preeminent species within the cancer microbiome, exhibiting superior virulence in comparison to other *Candida* species within its host (Narunsky-Haziza et al., 2022; Wang et al., 2023).

Per scholarly investigations, the principal component of the cell wall of *C. albicans*, a fungus frequently identified as white sponge nevus, is the yeast-derived polysaccharide, *zymosan*. Fascinatingly, it has been disclosed that *zymosan* possesses the capacity to induce the adhesion of oral cancer cells, thereby facilitating their proliferation. Moreover, recent elucidations have unveiled the presence of a newly discovered virulence factor *candidalysin*, encoded by the ECE1 gene within *C. albicans.* This *candidalysin* exhibits the capability to inflict damage upon epithelial cells and to activate multiple signaling pathways including MAPK and EGFR, which are recognized as contributors to cancer progression (Wang et al., 2023).

Additionally, among cancer patients, the concurrent manifestation of fungal and bacterial co-infections is a noteworthy phenomenon. Literature suggests that the synergistic interactions between bacteria and *C. albicans* may potentiate the upregulation of oncogenes, including PI3KCA and hRAS, thus amplifying the pro-oncogenic capacities of *C. albicans* (Amaya Arbeláez et al., 2021). Consequently, the contribution of *C. albicans* to cancer progression appears to be mediated through a confluence of factors: the formulation of carcinogenic metabolites, provocation of chronic inflammation, reconfiguration of the immune microenvironment, initiation of pro-oncogenic signaling cascades, and symbiotic interactions with bacterial counterparts (MacAlpine et al., 2023).

Several investigations suggest that oral leukoplakia and oral lichen planus represent premalignant lesions of oral cancer, which may implicate the involvement of *C. albicans* in their genesis (Sanjaya et al., 2011; Artico et al., 2014; Werneck et al., 2015; Dilhari et al., 2016; Stasiewicz and Karpiński, 2022). *C. albicans* is known to invigorate tumor metabolism and signaling pathways, in addition to amplifying the expression of metastatic markers, thereby facilitating the progression of oral cancer and modulating the carcinogenic landscape of the oral cavity (Vadovics et al., 2021; Wang et al., 2022).

It is noteworthy that previous research has documented a progressively increasing risk of oral and throat cancers in patients with *Candida* infections in recent years (Nørgaard et al., 2013; Vázquez-Olvera et al., 2023). Recent evidence establishes that there is a correlation between *Candida albicans* and cancer, notably with *Candida albicans* emerging as the primary pathogen linked to oral cancer in clinical settings.

#### 4.2.3 Treatment

From the perspective of keyword analysis, the enhancement of antifungal and anticancer therapeutic efficacies remains a pivotal area of ongoing research. Many cancer patients exhibit compromised immune systems due to frequent chemotherapy, leading to an increased susceptibility to fungal infections and an emerging resistance to certain antifungal medications, which significantly complicates the management of fungal infections. The relationship between *Candida albicans* and oral cancer is considered exceptionally close, as recurrent oral *Candida* infections and oral ulcers are among the manifestations of this malignancy (Stoopler et al., 2024). *Candida albicans* adheres to oral mucosa and forms biofilms, thus inducing a yeast-to-hypha transition, ultimately penetrating oral epithelial cells and inflicting long-term tissue damage, which elicits chronic inflammatory responses (Akpan and Morgan, 2002; Lass-Flörl et al., 2024). Microbial infection-induced chronic inflammation is acknowledged as a significant risk factor for tumor progression. Consequently, this phenomenon heightens the incidence of oral cancer and diminishes patient quality of life.

Contemporary research is chiefly oriented towards determining whether anticancer treatments alter the invasiveness of *Candida albicans* in the context of oncology. It is well-documented that host immune defenses become compromised upon the onset of cancer, consequently undermining the integrity of mucosal barrier structures and facilitating *Candida albicans* invasion. Prior investigations have demonstrated that anticancer therapies may mitigate the virulence factors of *Candida albicans*, notably the inhibition of biofilm formation, thereby diminishing its invasiveness (Wakharde et al., 2018; Yu and Liu, 2022). However, the facilitation of oral cancer progression by *Candida albicans* is mediated through complex and multifaceted interactive mechanisms, potentially exhibiting synergistic effects with the host. Consequently, the scope of present anticancer and antifungal therapies is relatively constrained, necessitating the exploration of innovative treatment strategies to alleviate clinical symptoms of *Candida albicans* infections in cancer patients, reduce the likelihood of tumor occurrence, and decelerate cancer progression.

### 4.3 Limitation

To guarantee a comprehensive and detailed literature analysis, the current investigation utilized the WoSCC as its primary source for information retrieval. Nevertheless, due to the rigorous stipulations and guidelines dictated by bibliometric analysis tools, this research initiative harbors certain limitations. Foremost, solely journal articles catalogued within the core collection of WoSCC were scrutinized to uphold data integrity. However, the potential for omissions within the database could introduce a minor degree of incompleteness to the data analysis. Nonetheless, this does not detract from the overarching trends delineated in this study. Secondly, an intrinsic element of subjectivity pervades quantitative analysis. Citation metrics adhere to temporal dependencies; thus, the deferred incorporation of latter high-caliber research articles into the database might exclude them from this specific analysis. Bibliometric approaches fall short in evaluating the singular quality of a research article. Nevertheless, despite these constraints, the outcomes of this inquiry still furnish valuable perspectives on the research focal points and trends linking *C. albicans* and cancer, thereby endowing researchers in correlated disciplines with a significant point of reference.

## 5 Conclusion

Within the scope of this analysis, we executed a bibliometric evaluation employing VOS Viewer and CiteSpace to assess the scholarly output of various nations, academic institutions, authors, journals, and research domains. The consistent augmentation in the volume of annual scholarly publications denotes an escalating global interest in this domain. While the United States, China, and India exhibit substantial volumes of publication output, there remains potential for amplification in the scholarly contributions of other nations. Moreover, cultivating intimate international collaborations would confer benefits in unveiling new mechanistic insights. This approach not only enhances the diversity of perspectives but also galvanizes the collective intellectual acumen necessary to advance understanding in the multifaceted intersections of *C. albicans* and cancer research.

Our investigational outcomes illuminate a multitude of contemporaneous research emphases—encompassing the nexus between *C. albicans* and cancer, the interaction of *C. albicans* with oral malignancies, and the realm of antifungal and antineoplastic treatments. In summarization, this inquiry furnishes foundational insights for academics engrossed in this field. Despite the nascent corpus of research on the correlation between *C. albicans* and cancer, attributable to the complexities inherent in their interplay and the challenges posed by antifungal therapy in oncological contexts, we maintain a conviction that continued investigation into this phenomenon will flourish with vigor. Such lines of inquiry possess critical research import and harbinger auspicious prospects for delineating targets within anti-cancer therapeutic strategies, potentially ushering in a new epoch of medical interventions that harmonize antifungal and anticancer paradigms.

## Data availability statement

Publicly available datasets were analyzed in this study. This data can be found at: https://www.webofscience.com/wos/woscc/summary/df33afba-f843-41e8-b932-cb3678eb8243-e92e7316/relevance/1.

## Author contributions

ST: Conceptualization, Data curation, Formal analysis, Investigation, Methodology, Software, Validation, Writing – original draft, Writing – review & editing. YX: Data curation, Formal analysis, Supervision, Validation, Writing – review & editing. XL: Funding acquisition, Investigation, Project administration, Resources, Supervision, Writing – review & editing.
